# Resective, Ablative and Radiosurgical Interventions for Drug Resistant Mesial Temporal Lobe Epilepsy: A Systematic Review and Meta-Analysis of Outcomes

**DOI:** 10.3389/fneur.2021.777845

**Published:** 2021-12-09

**Authors:** Kajol Marathe, Ali Alim-Marvasti, Karan Dahele, Fenglai Xiao, Sarah Buck, Aidan G. O'Keeffe, John S. Duncan, Vejay N. Vakharia

**Affiliations:** ^1^Department of Clinical and Experimental Epilepsy, University College London, London, United Kingdom; ^2^National Hospital for Neurology and Neurosurgery, London, United Kingdom; ^3^Wellcome/EPSRC Centre for Interventional and Surgical Sciences, University College London, London, United Kingdom; ^4^Department of Statistical Science, University College London, London, United Kingdom

**Keywords:** surgery, epilepsy, MTLE = mesial temporal lobe epilepsy, LITT = laser interstitial thermal therapy, radiosurgery, radiofrequency ablation (RFA)

## Abstract

**Objectives:** One-third of individuals with focal epilepsy do not achieve seizure freedom despite best medical therapy. Mesial temporal lobe epilepsy (MTLE) is the most common form of drug resistant focal epilepsy. Surgery may lead to long-term seizure remission if the epileptogenic zone can be defined and safely removed or disconnected. We compare published outcomes following open surgical techniques, radiosurgery (SRS), laser interstitial thermal therapy (LITT) and radiofrequency ablation (RF-TC).

**Methods:** PRISMA systematic review was performed through structured searches of PubMed, Embase and Cochrane databases. Inclusion criteria encompassed studies of MTLE reporting seizure-free outcomes in ≥10 patients with ≥12 months follow-up. Due to variability in open surgical approaches, only comparative studies were included to minimize the risk of bias. Random effects meta-analysis was performed to calculate effects sizes and a pooled estimate of the probability of seizure freedom per person-year. A mixed effects linear regression model was performed to compare effect sizes between interventions.

**Results:** From 1,801 screened articles, 41 articles were included in the quantitative analysis. Open surgery included anterior temporal lobe resection as well as transcortical and trans-sylvian selective amygdalohippocampectomy. The pooled seizure-free rate per person-year was 0.72 (95% CI 0.66–0.79) with trans-sylvian selective amygdalohippocampectomy, 0.59 (95% CI 0.53–0.65) with LITT, 0.70 (95% CI 0.64–0.77) with anterior temporal lobe resection, 0.60 (95% CI 0.49–0.73) with transcortical selective amygdalohippocampectomy, 0.38 (95% CI 0.14–1.00) with RF-TC and 0.50 (95% CI 0.34–0.73) with SRS. Follow up duration and study sizes were limited with LITT and RF-TC. A mixed-effects linear regression model suggests significant differences between interventions, with LITT, ATLR and SAH demonstrating the largest effects estimates and RF-TC the lowest.

**Conclusions:** Overall, novel “minimally invasive” approaches are still comparatively less efficacious than open surgery. LITT shows promising seizure effectiveness, however follow-up durations are shorter for minimally invasive approaches so the durability of the outcomes cannot yet be assessed. Secondary outcome measures such as Neurological complications, neuropsychological outcome and interventional morbidity are poorly reported but are important considerations when deciding on first-line treatments.

## Introduction

Despite optimal anti-seizure medication treatment, about one-third of individuals with epilepsy still suffer from seizures. If the seizure onset zone is accurately delineated during presurgical evaluation, surgery can result in sustained seizure freedom in patients with drug resistant focal epilepsy (1). The first randomized control trial of surgery for temporal lobe epilepsy showed seizure freedom rates of 58% in patients randomized to surgery compared to 8% randomized to best medical therapy at 12 months (2). Additional benefits of surgery include improved quality of life, cognitive performance, and minimizing risk of sudden unexpected death in epilepsy (SUDEP) (3). In this study, anteromesial temporal lobe resections (ATLR) were performed through a lateral neocortical resection of 4–4.5 cm in the dominant hemisphere and 6–6.5 cm in the non-dominant hemisphere as measured from the temporal pole followed by amygdala and 1–3 cm hippocampal resection (2). Since then a number of different surgical approaches and modifications have been implemented including minimizing the lateral neocortical resection to 3 cm, sparing of the superior temporal gyrus (4) and various selective approaches including transcortical (5), trans-sylvian (6) and subtemporal (7) amygdalohippocampectomy.

Each of these surgical modifications were undertaken with the aim of minimizing the neurological morbidity and neuropsychological sequelae secondary to collateral damage of nearby structures (8). Results of selective approaches have been variable with some studies showing improved neuropsychological outcomes and others showing worse seizure freedom rates than ATLR (9). Open surgical procedures all have inherent risks including visual field deficit, memory decline, stroke, hemorrhage and infection (10). More recently, novel “minimally invasive” techniques have been introduced with the aim of further reducing collateral injury and averting the need for craniotomy, including radiosurgery (SRS) (11), radiofrequency ablation (RF-TC) (12) and laser interstitial thermal therapy (LITT) (13). As with other novel procedures, early results have been mixed, principally due to small study sizes, short follow-up durations and as the technology matured. The lack of high-quality comparative studies has made interpretation difficult (14). Coupled with this is the effect of the learning curve, whereby outcomes continue to improve as health-care systems gain experience in selecting patients for and performing these novel procedures. The introduction of novel technologies also has the potential to expose patients to additional harm until comparative long-term outcomes are known.

The objective of this study was to undertake a “Preferred Reporting Items for Systematic Reviews and Meta-Analysis” (PRISMA) systematic review and meta-analysis (15) of all ablative methods for the treatment of drug resistant mesial temporal lobe epilepsy.

## Methods

### Eligibility Criteria

Eligibility for inclusion in the meta-analysis include peer-reviewed publications in which full length English language manuscripts were available through electronic indexing comprising: a) clinical studies of patients with temporal lobe epilepsy, b) undergoing open epilepsy surgery as a treatment, or c) undergoing RF-TC, SRS or LITT as a treatment, d) with greater than 10 patients in the intervention arm and e) follow-up duration of ≥12 months. Due to previous meta-analyses of open surgery outcomes (16, 17) only comparative open surgical studies were included to provide the highest levels of evidence. Studies that did not report Engel or ILAE outcomes or similar were also excluded.

### Information Sources

Using the PRISMA guidelines (15) a structured search of the PubMed, Embase and Cochrane databases were undertaken. The last date of the search was September 9^th^ 2020.

### Search Strategy

Two independent researchers (VNV and FX) applied the search criteria defined following a PICOS (participants, interventions, comparators, outcomes and study design) approach to identify search terms. Participants included human studies reporting surgical treatments for drug resistant focal epilepsy. Surgical interventions included in the search terms were open surgical techniques including selective approaches and the numerous variations, as well as minimally invasive alternatives such as LITT, RF-TC, SRS and high frequency ultrasound. The main comparator for the quantitative analysis was seizure-free outcome.

The search terms can be found in the Supplementary Information.

The reference lists of all studies were searched and cross-references for additional eligible studies considered. Previous systematic reviews and meta-analyses were also screened to capture additional studies (9, 16–18) not identified by the search terms.

### Outcomes

During full text review data extraction was performed using a table with a predefined set of criteria including level of evidence, study design, comparison/control group and sample size. At a participant level data relating to side of surgery, patient age, duration of epilepsy, pre-operative MRI findings, post-operative histological findings, follow up duration, seizure outcome grading scale, post-operative seizure outcome, independent predictors of surgical outcome, neuropsychological tests performed, neuropsychological outcome, psychiatric outcome, operative morbidity and operative mortality was recorded. The main outcome for meta-analytic comparison was seizure-free outcome.

### Risk of Bias

Anticipated sources of bias affecting seizure freedom included the seizure outcome grading scale and the duration of follow-up. To mitigate outcome reporting for different grading scales only comparable seizure outcomes were included in the quantitative analysis and where sufficient information was provided outcomes were converted from one grading scale to another; e.g., Engel class 1(A-D) = ILAE class 1, 1a and 2. The issue of overlapping patient cohorts in different papers also required careful analysis of the subjects so that duplicate cohorts were not included in the quantitative analysis. Where duplicate cohorts were unclear, authors were contacted for clarification.

### Appraisal of Evidence

Methodological Index for Non-Randomized Studies (MINORS) (19) and Jadad (20) scores for randomized control trials, respectively, were calculated independently by KM and KD. Low scores suggest less methodologically sound studies.

### Synthesis of Results

For each surgery type, pooled estimates of the seizure-free rate per person-year were calculated, together with 95% confidence intervals, using a meta-analysis with inverse variance weighting.

The seizure-free rate per person-year estimates were calculated using the following equation to allow for the varying follow up durations between studies. As all patients are seizure-free immediately following intervention the time at risk of seizure-relapse can be taken as the total follow-up duration of the study with Person-years representing the sum of the total time at risk (total number of patients x follow up duration of study). This approach allows studies with differing follow-up times to be included in the meta-analysis and yields an informative method to compare studies having accounted for follow-up duration. In contrast, restricting the analysis to include only studies where follow-up is of the same length may result in selection bias and loss of information.


$$\begin{array}{l} \text{Seizure free rate per person year}\\ =\frac{\text{Number of patient years seizure free}}{\text{Total number of patient years at risk}} \end{array}$$

A mixed-effects linear regression model was then performed with rate of seizure freedom per person-year and surgery method as a covariate to compare seizure-free rates between surgery types. A random effect for study was included in this model. Statistical analyses were performed using R (version 4.10).

## Results

**Figure 1:**
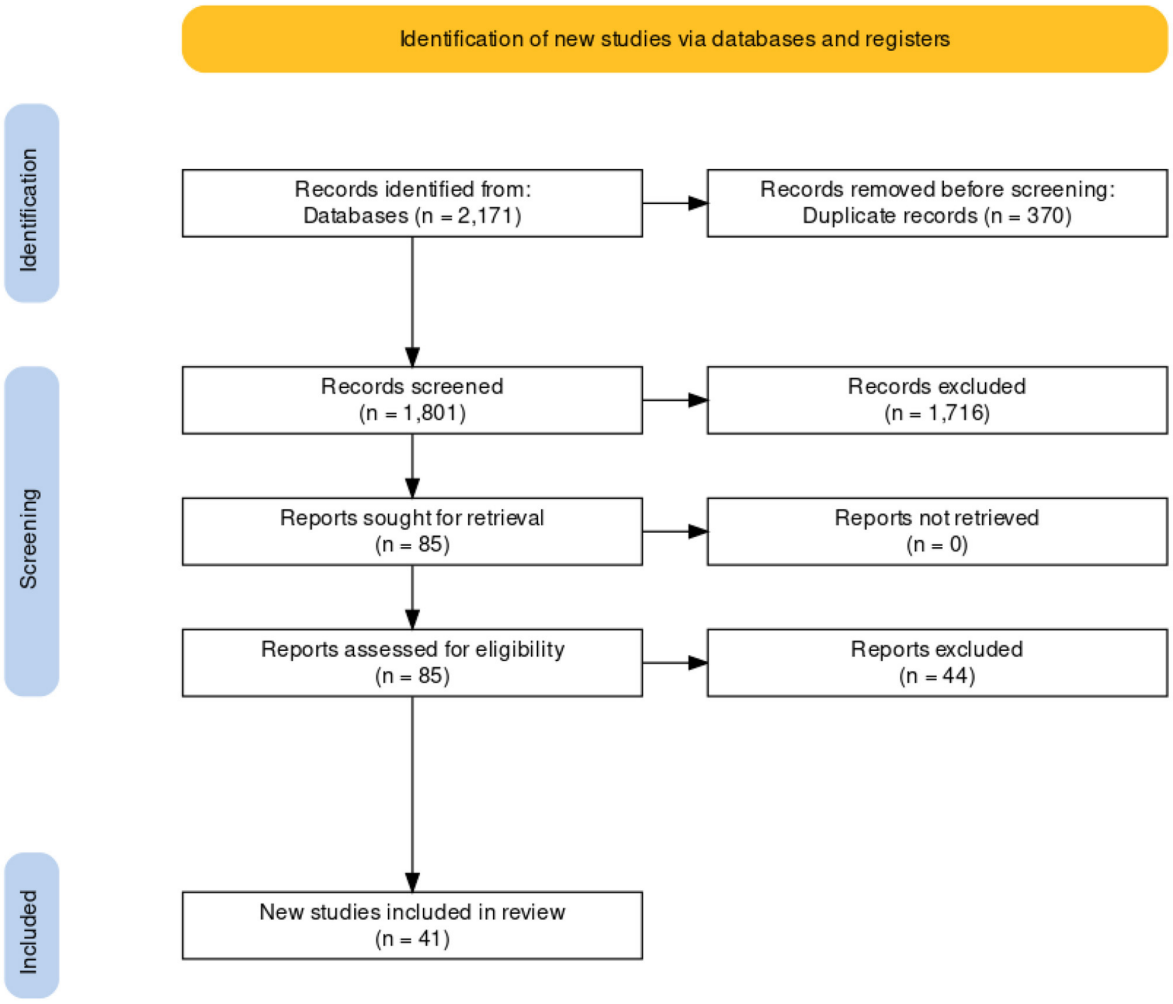
PRISMA flowchart.

In total, 2,171 studies were initially identified across three different databases. Following removal of duplicate and non–English-language studies 1,801 manuscript titles and abstracts were screened. After applying the eligibility criteria, 85 underwent full text review. Any discrepancies between eligible publications were resolved by the senior author. From the full text review, 41 studies (19 on open surgery, 11 on LITT, four on radiofrequency and seven on radiosurgery) were included in the quantitative synthesis (Figure 1). No eligible studies were found on ultrasound. It was not possible to conduct a meta-analysis for neuropsychological outcomes or complications as no standardized tests or reporting criteria were adhered to.

### Open Surgery

Where provided, patient demographics regarding side of resection, age at operation, and duration of epilepsy were comparable between open surgical interventions (Table 1). Overall, 86.5% of patients had MTS on pre-op MRIs and 79.5% had histologically proven MTS from 14 studies.

**Table 1:** Open surgery patient characteristics.

**Type of intervention**	**Side of resection**	**Age in years (mean)**	**Duration of epilepsy in years (mean)**
ATLR	206 R 225 L	33 (from nine studies)	25.5 (from 10 studies)
SAH (transcortical)	170 R 188 L	35.1 (from 9 studies)	24.6 (from nine studies)
SAH (transsylvian)	39 R (from two studies) 39 L (from two studies)	34.1 (from three studies)	25.4^*^

* *[only stated in Wendling et al. (21)]*.

Nineteen studies comparing open surgical outcomes were included in the meta-analysis (Table 2). ATLR was compared to transcortical SAH in 63% (12/19), to trans-sylvian SAH in 26% (5/19) and to an unspecified SAH technique in 11% (2/19). Of the studies comparing ATLR with transcortical SAH, 25% (3/12) were prospective whilst the remaining 75% (9/12) were retrospective. One pilot RCT compared ATLR with transcortical SAH and transcortical parahippocampal gyrus resection (22). The study sample size was small and being a pilot study was underpowered to detect differences in Engel 1a outcome between any of the three techniques at one- and five-year follow-up. Despite this, visual field defects were noted to be significantly worse in the ATLR and SAH groups compared to the parahippocampectomy group.

**Table 2:** Open surgery.

**Authors**	**Publication Year**	**Type of study**	**Method**	**Comparator**	**Number of patients followed up**	**Duration of follow up**	**Seizure free rate**	**MINORS/** **JADAD score**
Bate et al.	2007	Retrospective	ATLR	Transcortical SAH	ATLR: 82	12 months	ATLR: 62% (51/82)	16/24
					SAH: 32		SAH: 34% (11/32)	
Bujarski et al.	2013	Retrospective	ATLR	Transcortical SAH	ATLR: 30	60 months	ATLR: 77% (23/30)	15/24
					SAH: 39		SAH: 69% (27/39)	
Mansouri et al.	2014	Retrospective	ATLR	Transcortical SAH	ATLR: 75	24 months	ATLR: 51% (38/75)	15/24
					SAH: 21		SAH: 43% (9/21)	
Nascimento et al.	2016	Retrospective	ATLR	Transcortical SAH	ATLR: 22	60 months	ATLR: 63.6% (14/22)	18/24
					SAH: 23		SAH: 73.9% (17/23)	
Paglioli et al.	2006	Prospective	ATLR	Transcortical SAH	ATLR: 80	ATLR: 80.4 months	ATLR: 91.3% (73/80)	14/24
					SAH: 81	SAH: 54 months	SAH: 86.4% (70/81)	
Sagher et al.	2012	Retrospective	ATLR	Transcortical SAH	ATLR: 51	ATLR: 43.2 months	ATLR: 92.2% (47/51)	15/24
					SAH: 45	SAH: 44.7 months	SAH: 95.6% (43/45)	
Tanriverdi et al.	2008	Retrospective	ATLR	Transcortical SAH	ATLR: 50	60 months	ATLR: 64% (32/50)	17/24
					SAH: 50		SAH: 64% (32/50)	
Tanriverdi et al.	2010	Retrospective	ATLR	Transcortical SAH	ATLR: 123	12 months	ATLR: 65.9% (81/123)	16/24
					SAH: 133		SAH: 58.6% (78/133)	
Elliott et al.	2018	Retrospective	ATLR	Transcortical SAH	ATLR: 61	63.6 months	ATLR: 77% (47/61)	17/24
					SAH: 18		SAH: 44% (8/18)	
Mohan et al.	2018	Retrospective	ATLR	Transcortical SAH	ATLR: 178	60 months	ATLR: 49% (87/178)	16/24
					SAH: 37		SAH: 31% (11/37)	
Foged et al.	2018	Prospective	ATLR	Transcortical SAH	ATLR: 34	12 months	ATLR: 73.5% (25/34)	18/24
					SAH: 22		SAH: 72.7% (16/22)	
Arturo Alonso-Vanegas et al.	2018	Prospective RCT	ATLR	Transcortical SAH	ATLR: 14	60 months	ATLR: 64.3% (9/14)	3/5
					SAH: 15		SAH: 66.7% (10/15)	
Clusmann et al.	2002	Retrospective	ATLR	Transsylvian SAH	ATLR: 98	38 months	ATLR: 69% (68/98)	16/24
					SAH: 138		SAH: 70% (96/138)	
Lee et al.	1997	Retrospective	ATLR	Transsylvian SAH	ATLR: 25	12 months	ATLR: 60% (15/25)	17/24
					SAH: 13		SAH: 38.5% (5/13)	
Morino et al.	2006	Retrospective	ATLR	Transsylvian SAH	ATLR: 17	12 months	ATLR: 71% (12/17)	16/24
					SAH: 32		SAH: 78% (25/32)	
Schramm et al.	2011	Prospective	ATLR	Transsylvian SAH	ATLR: 74	12 months	ATLR: 83.8% (62/74)	5/5
					SAH: 125		SAH: 67.2% (84/125)	
Wendling et al.	2013	Retrospective	ATLR	Transsylvian SAH	ATLR: 49	84 months	ATLR: 85.7% (42/49)	18/24
					SAH: 46		SAH: 78.3% (36/46)	
Arruda et al.	1996	Retrospective	ATLR	Unspecified SAH	ATLR: 37	12 months	ATLR: 68% (25/37)	17/24
					SAH: 37		SAH: 76% (28/37)	
Mackenzie et al.	1997	Retrospective	ATLR	Unspecified SAH	ATLR: 72	12 months	ATLR: 60% (43/72)	15/24
					SAH: 28		SAH: 21% (6/28)	

One study, an RCT, reported the SAH group having a significantly lower seizure free outcome (*p* = 0.013) at 12 months (23). The remaining four studies did not report any significant differences between the two techniques including one paper (24) which was a subset of patients from Mackenzie et al. (25). Two studies did not specify the type of SAH performed so were excluded from the quantitative analysis for SAH (25, 26). One study evaluated a pediatric cohort (mean age 10.6 years) and found that ATL had a significantly better seizure outcome (*p* = 0.017) after a mean of 5.3 years (27). Studies comparing ATLR with radiosurgery (11) and with radiofrequency (28) were also included in the meta-analysis. In summary, of all the comparative open surgical techniques, two studies provided class one evidence (22, 23), one class two (29) and the remainder class three. A total of 2,183 patients were included in the quantitative synthesis with 1,248 undergoing ATLR and 935 undergoing SAH (transcortical 516, trans-sylvian 354 and unspecified 65).

**Figure 2:**
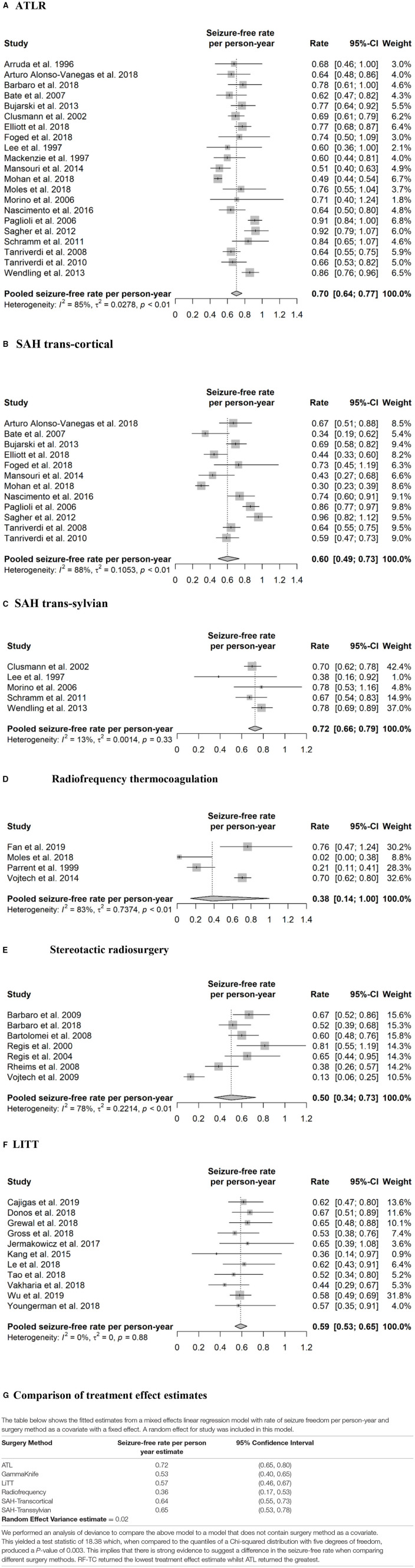
Forest plots of effect sizes and 95% confidence intervals. **(A)** ATLR. **(B)** SAH trans-cortical. **(C)** SAH trans-sylvian. **(D)** Radiofrequency thermocoagulation. **(E)** Stereotactic radiosurgery. **(F)** LITT. **(G)** Comparison of treatment effect estimates.

Meta-analysis revealed a pooled effect size for overall seizure free rate per-person year of 0.70 (95% CI 0.64–0.77) for ATLR (Figure 2A), 0.60 (95% CI 0.49–0.73) for transcortical selective amygdalohippocampectomy (Figure 2B) and 0.72 (95% CI 0.66–0.79) for trans-sylvian selective amygdalohippocampectomy (Figure 2C).

Neuropsychological outcome was reported in 16 studies. Five comparative open surgery studies investigated changes in intellectual status after surgery. Across these studies, intelligence either remained stable (27, 30, 31) or improved in both the visuospatial and verbal domain (32, 33). The improvement occurred as a combined effect of side and type of intervention, with mixed findings across studies. No post-surgical decline in intelligence was found.

Four studies investigated language functions. Language was reported to either remain stable (30, 31) or decline (34, 35) after surgery, but no study reported post-surgical improvement. Decline in language abilities were observed irrespective of side or procedure (35) with greatest declines reported following left-sided resections and ATLR (34).

Nine studies which looked at open surgery only investigated visuospatial memory and 13 investigated verbal memory. Four studies reported no post-operative changes in visuospatial memory whereas for verbal memory, just over half of studies suggested a decline (21, 24, 29, 30, 33, 35, 36). Post-operative improvements in memory are reported for functions subserved by the non-operated side, with improvements in visuospatial memory occurring after left-sided resections (33, 34) and verbal memory gains after right-sided resections (32). Intervention-specific improvement of memory is also observed, with greater improvement after SAH than after ATLR, particularly for visuospatial memory (21, 33, 34). Visuospatial memory also declined after right-sided resection, irrespective of the intervention type (34) or with greater decline after ATLR compared to SAH (33). The factors influencing verbal memory decline varied across studies, with either a decline irrespective of side or type of resection (35), after left-sided resection irrespective of the intervention type (21, 29), or conflicting findings regarding the effect of intervention [i.e. with more decline after SAH (30, 33) or ATLR (24, 29, 36)].

#### Complications

Data regarding post-operative complications were missing in 8/19 of the comparative open surgical studies, with one study only reporting cognitive and psychiatric complications (31). Even in those that did report complications, reporting was often not comprehensive. Complications reported here are described for the open surgical techniques as reliable differentiation between SAH and ATLR was not possible. The two studies that compared ATLR with radiofrequency and radiosurgery are also included here for the ATLR arm only. Of the studies that reported complications, the most common complication was visual field defects, occurring in 4% (63/1,548) of patients. This is much lower than complications reported for open surgery where formal perimetry was utilized (37) and because of non-recognition of such defects in many studies. Most studies did not use perimetry and only reported clinically significant visual defects. Cranial nerve palsy rate was 0.78% (12/1548) and 1.29% (20/1548) suffered from infection-related complications including meningitis, mastoiditis and one case of osteomyelitis. From all identified studies mortality was 0.82% (18/2,183) over a period of 5 (38) to 7 (29) years post-surgery.

Psychiatric complications such as anxiety, depression and psychosis were reported in 1.1% (17/1,548) patients, however this was only across the four studies that reported such outcomes. Therefore, this will be a gross underestimate as many studies did not report psychiatric outcomes. Neurological complications were reported in 1.87% (29/1,548) patients and included dysphasia, dysnomia, hemiparesis, hemiplegia, hypoacusis and cerebritis. Other complications included CSF leaks or collections (10/1,548), pseudomeningoceles (4/1,548), haematoma (9/1,548), jaw pain (4/1,548), pulmonary embolism (2/1,548), venous thrombosis (5/1,548) and ventilator associated pneumonia (1/1,548). Two papers did not provide a full breakdown (23, 34), but reported neurological complications in 3–5.2%, overall surgical complication rate as 8.5% (34) and permanent morbidity as 1.67%. Overall, the heterogeneity in reported complications was difficult to synthesize, but overall mortality and morbidity was relatively low.

#### Risk of Bias

The median MINORS score for comparative studies was 16/24, indicating that most studies contained methodological flaws and may therefore contribute a moderate degree of bias. One RCT returned a JADAD score of 3/5 (22), indicating a moderate degree of bias, whilst another RCT scored 5/5 (23).

### Radiofrequency Thermocoagulation

Four studies were included in the quantitative analysis (12, 28, 39, 40). Where reported, the mean age was 33.6 years and the mean duration of epilepsy was 20.3 years, with some studies reporting median ages only. 64 patients underwent left-sided procedures, and 37 patients underwent right-sided procedures. Laterality was not reported in one study (28). There was unclear reporting of the exact number of patients with MTS on MRI, however it was present in the majority. Histopathological confirmation of MTS was not available as this is a lesioning technique.

One study (39) was prospective, one study did not specify (12), and the remainder were retrospective. The range of reported seizure freedom was between 0 and 76.2% seizure free at 12 months (Table 3). An single study which followed an overlapping patient cohort with Vojtech et al. (40), compared RF-TC to ATLR and reported no difference in Engel 1 outcomes of 79.3% (23/29) and 76.5% (13/17) at 5 year follow up, respectively (41). In contrast, a later study comparing RF-TC to ATLR (28), reported none (0/21) of the patients in the RF-TC group were seizure free whereas 75.5% (37/49) of the patients in the ATLR group had an Engel 1a outcome at 12-month follow up (*p* < 0.0001). Overall, 122 patients from studies equating to level two, three and four evidence were included in the quantitative synthesis. A random-effects meta-analysis yielded a pooled seizure free rate estimate per person-year of 0.38 (95% CI 0.14–1.00) (Figure 2D).

**Table 3:** Radiofrequency.

**Authors**	**Publication** **Year**	**Type of study**	**Method**	**Comparator**	**Number of patients followed up**	**Duration of follow up**	**Seizure free rate**	**MINORS/JADAD score**
Parrent et al.	1999	Unclear	Radiofrequency	N/A	19	25.8 months	22% (4/19)	9/16
Vojtech et al.	2014	Retrospective	Radiofrequency	N/A	61	63.6 months	70.5% (43/61)	10/16
Fan et al.	2019	Prospective	Radiofrequency	N/A	21	12 months	76.2% (16/21)	11/16
Moles et al.	2018	Retrospective	Radiofrequency	ATLR	Radiofrequency: 21	12 months	Radiofrequency: 0% (0/21)	16/24
					ATLR:49		ATLR: 75.5% (37/49)	

Neuropsychological outcome was reported in one of four studies. This single study suggested better post-operative neuropsychological outcome in the RF-TC group (41) compared with the ATLR group.

Complications were heterogenous across the studies. 4.1% (5/122) patients had haematomas, which were mostly asymptomatic (12, 40). Other complications included transitory anosmia (1/122), upper quadrantanopia (1/122), meningitis (2/122), pulmonary embolism in a patient with hereditary coagulopathy (1/122), and an asymptomatic retention of an electrode fragment (2/122). No complications or mild headache were reported in 36.1% (44/122) patients (40). The most serious early complications were a small intracerebral haematoma and hydrocephalus which resolved with ventricular drainage (40). Reported mortality was 5% (3/61) in the report by Vojtech et al. (40), due to one suicide, an unrelated accident and extracranial malignancy. Mortality was 0% in all other studies. No mortality was directly related to the operative procedure. Psychiatric complications were reported in 6/61 patients (40).

#### Risk of Bias

The median MINORS score for radiofrequency was 10/16 for non-comparative studies and 16/24 for a single comparative study indicating a moderate risk of bias due to methodological flaws.

### Stereotactic Radiosurgery

Seven studies (three prospective) comprising 133 patients were included in the meta-analysis (Table 4). Two patients in the paper by Rheims et al. (42) and six patients in the paper by Vojtech et al. (43) overlapped with those of the Marseilles cohort (44) and were excluded from the analysis. The mean duration of epilepsy was similar between studies, with an overall mean of 24.1 years. Between 93 and 100% had MTS on MRI scan. Where laterality was reported, 48 procedures were left-sided and 40 were on the right.

**Table 4:** Stereotactic radiosurgery.

**Authors**	**Publication Year**	**Type of study**	**Method**	**Comparator**	**Number of patients followed up**	**Duration of follow up**	**Seizure free rate**	**MINORS/** **JADAD score**
Barbaro et al.	2009	Prospective RCT (low dose vs high dose)	Gamma Knife	Low dose vs high dose	30	36 months	67% (20/30)	3/5
Barbaro et al.	2018	Prospective RCT (ROSE trial)	Gamma Knife	ATLR	Gamma Knife: 31	36 months	Gamma Knife: 52% (16/31)	3/5
					ATLR: 27		ATLR: 78% (21/27)	
Bartolomei et al.	2008	Retrospective	Gamma Knife	N/A	15	96 months	60% (9/15)	9/16
Regis et al.	2000	Retrospective	Gamma Knife	N/A	16	24 months	81% (13/16)	8/16
Regis et al.	2004	Prospective	Gamma Knife	N/A	20	24 months	65% (13/20)	11/16
Rheims et al.	2008	Retrospective	Gamma Knife	N/A	13	60 months	38.5% (5/13)	10/16
Vojtech et al.	2009	Retrospective	Gamma Knife	N/A	8	91.5 months	12.5% (1/8)	9/16

Due to the delay in treatment benefit following SRS, seizure free outcomes were reported from 24 months. All studies reported on the use of the gamma knife (Elekta AB, Sweden). One study compared high (24Gy) and low (20Gy) dose SRS, reporting improved seizure free outcomes but alongside increased minor complications associated with use of the higher dose (45). The highest level of evidence was from the ROSE randomized control trial comparing SRS to ATLR (11). The trial was terminated early due to poor recruitment, but even at this stage revealed that seizure free outcome rates following ATLR were superior to SRS (*p* = 0.82 at 15% non-inferiority margin). Most studies provided level three and four evidence with two papers providing level two evidence (44, 45) and a single RCT providing level one evidence (11). The pooled seizure-free rate per person-year was 0.50 (95% CI 0.34–0.73) after a treatment delay of 24 months (Figure 2E). Neuropsychological outcome was reported in 5/7 studies. One study directly compared outcome between SRS and open surgery (ATLR) and showed less risk of verbal memory deterioration after SRS, and less risk of visuospatial memory deterioration after ATLR (11). Another neuropsychological study reported decline in verbal memory with high dose (24 Gy) but not low dose (20 Gy) Gamma Knife to the dominant amygdalohippocampal complex (46). Other studies either reported some improvement in a subset of the patients or little post-operative cognitive change.

#### Complications

Mortality was 1.5% (2/133) owing to one case of SUDEP (47) and one case of right cerebellar hemorrhage unrelated to the surgery (42). The classification of what defined “serious” adverse effects were not standardized throughout studies. Additionally, definitions of “early” and “late” complications were not specified. Cerebral oedema was reported in 7.5% (10/133) of patients undergoing SRS. One case of serious cerebral oedema not responding to dexamethasone required temporal lobectomy (45). Interestingly, Bartolomei (47) did not report cerebral oedema in any of their patients. Corticosteroid treatment was needed in 47.4% (63/133) when excluding overlapping patients. Other side effects included headaches (24/133), pin-site infection (1/133), dizziness (2/133), nausea (2/133), vomiting (2/133), serious seizure exacerbation (2/133), status epilepticus (2/133) and visual field defects in the realm of 35.3% (47/133) when excluding overlapping patients' results. In one study, 93% of those formally tested had some form of visual field defect (37), even though only 10 were reported to have a superior quadrantanopia. Vojtech et al. (48) reports an even-longer term mean follow up time of 16 years of the complications from Vojtech et al., stressing the need for life-long MRI follow up in patients undergoing gamma knife radiosurgery, as delayed radio-necrosis and cyst formation were reported. Two patients developed psychosis (43) and one reported developing depression post-surgery (44).

#### Risk of Bias

For the two RCTs, JADAD scores were 3/5. A median MINORS score of 9/16 was scored for the remaining studies, all of which were non-comparative, indicating a moderate degree of bias within these studies.

### Laser Interstitial Thermal Therapy

Two studies (49, 50) reported duplicate patient cohorts and were excluded from the analysis. From the 11 studies reporting LITT outcomes, two were prospective (51, 52) and nine were retrospective (see Table 5). Overall, 520 patients were followed up, ranging between a mean of 12 months (13, 53–56) and 43 months (57). Engel 1 outcomes for the studies ranged between 44% (14) and 67.4% (58). The mean age of patients was 41.9 years, mean duration of epilepsy was reported in nine studies as 23.8 years, an average of 74.2% patients undergoing LITT had MTS on MRI, and the side of resection was reported by nine studies as 263 (left) and 201 (right).

**Table 5:** Laser interstitial thermal therapy.

**Authors**	**Publication Year**	**Type of study**	**Method**	**Number of patients followed up**	**Duration of follow up**	**Seizure free rate**	**MINORS/JADAD score**
Gross et al.	2018	Retrospective	LITT	58	12 months	53.4% (31/58)	10/16
Jermakowicz et al.	2017	Retrospective	LITT	23	12 months	65% (15/23)	10/16
Kang et al.	2015	Retrospective	LITT	11	12 months	36.4% (4/11)	10/16
Vakharia et al.	2018	Retrospective	LITT	25	24.4 months	44% (11/25)	11/16
Youngerman et al.	2018	Retrospective	LITT	30	12 months	57% (17/30)	12/16
Donos et al.	2018	Retrospective	LITT	43	20.3 months	67.4% (29/43)	9/16
Wu et al.	2019	Retrospective	LITT	231	12 months	58.0% (134/231)	10/16
Le et al.	2018	Prospective	LITT	29	18 months	62% (18/29)	11/16
Cajigas et al.	2019	Retrospective	LITT	26	42.9 months	61.5% (16/26)	11/16
Tao et al.	2018	Prospective	LITT	21	24 months	52% (11/21)	13/16
Grewal et al.	2018	Retrospective	LITT	23	34 months	65.2% (15/23)	10/16

A retrospective case series revealed that trajectories with a medial course through the hippocampal head resulted in less mesial hippocampal head remnant and was associated with improved seizure free outcome (53). Seizure freedom rates were found to be twice as high in patients with MTS compared to non-MTS (13), although a separate study reported similar seizure freedom rates between MTS and non-MTS when stereoelectroencephalography (SEEG) was utilized to prove mesial temporal lobe onset (55). Overall, using data from the 11 LITT studies included in the quantitative analysis a pooled estimate of the seizure-free rate per person-year was 0.59 (95% CI 0.53–0.65) (Figure 2F).

#### Neuropsychological Outcome

Neuropsychological outcomes were reported in six of the eleven studies. Amongst these, three investigated visuospatial memory and six investigated verbal memory. Only one directly compared post-operative neuropsychological outcomes between LITT and ATLR (50). This demonstrated that the ATLR group showed post-surgery decline in visuospatial memory, whereas those who underwent LITT remained stable. Other studies report a significant decline in verbal memory after LITT in the dominant hemisphere (53, 58) whilst others report significant decreases in verbal intelligence but an improvement in visuospatial intelligence in the dominant hemisphere (58). In contrast, Gross et al. (13) reported an improvement in delayed verbal recall for non-dominant procedures. With regards to language function, it remained overall stable, with a smaller decline in memory in non-dominant procedures (59) or improved, for both dominant and non-dominant procedures (50).

#### Complications

The reporting of complications varied across studies, with some distinguishing between asymptomatic and symptomatic visual field defects. Visual field dysfunction, including those that were transient and asymptomatic were reported in 7.5% (39/520) of patients; however, only a subset of patients had formal visual field testing. Cranial nerve palsies of the third and fourth cranial nerves were reported in 1.5% (8/520) patients, the majority of which were transient. Post-operative hemorrhage occurred in 0.96% (5/520) patients and aseptic meningitis occurred in 1/520. Other complications occurred in 7.31% (38/520). Mortality following LITT was 0.57% (three patients died: two from suicide, one from SUDEP).

### Psychiatric Outcome

The psychiatric complication rate was 4.4% (23/520), but this was only reported across five studies. Worsening of existing mood disorders was reported in 10 patients, worsening of mood in general was reported in two individuals and transient anxiety was reported in three. Two others needed hospitalization due to psychosis (14) or acute postoperative psychiatric complications (51), and five were diagnosed with a new onset affective disorder.

### Risk of Bias

The median MINORs score for LITT studies was 10/16, indicating a moderate degree of bias in the studies.

## Discussion

### Primary Outcome

The primary aim of this meta-analysis was to review the effectiveness of different interventional techniques for MTLE, by comparing seizure freedom rates for resective, ablative and SRS treatments. We were also interested in stereotactic radiosurgery as no meta-analysis to date has attempted to compare this to LITT, radiofrequency and open surgical methods. We also compared seizure free outcomes between ATLR, trans-sylvian and transcortical SAH. Due to heterogeneity within interventions, comparison within the random-effects model was not possible. However, a mixed-effects linear regression model suggested significant differences between interventions, with LITT, ATLR and SAH demonstrating the largest effects estimate and RF-TC the lowest (Figure 2G).

### Open Surgery

Between the open surgical methods, our mixed effects model for seizure-free rate per person year estimates were greatest for ATLR, followed by transsylvian SAH and then trans-cortical SAH, but overlap of the 95% confidence intervals prevents determination of comparative superiority. With regard to neuropsychological outcomes, open surgery does not impede on intellectual status and often even leads to post-operative improvement. Language ability, however, is prone to post-surgical decline, but the factors influencing such decline (such as the side and type of surgical intervention) are unclear. Whereas visuospatial memory is likely to either remain stable or improve after surgery, verbal memory is prone to post-surgical decline. In terms of the intervention type, SAH seems more beneficial than ATLR for visuospatial memory. On the other hand, mixed findings are reported regarding the effects of the type of surgical intervention on verbal memory. A meta-analysis of complications following ATLR reported psychiatric complications, visual field defects and cognitive disorders as most common (60). The fact that the efficacy and complications of open surgical techniques are so well-researched mean that it will require clear outcomes from minimally invasive techniques to replace surgical methods such as ATLR that have an established evidence base, including the results from this meta-analysis which provides further evidence for their efficacy in seizure control.

### Stereotactic Radiosurgery

The estimate of the seizure-free rate per person-year for those undergoing radiosurgery was lower than that for open surgical methods such as ATLR and transcortical SAH. Additionally, there were only seven papers (three prospective) included in the meta-analysis for radiosurgery as compared with the 21 papers that looked at ATLR. The highest level of evidence arises from the ROSE randomized control trial which revealed inferiority of SRS compared to ATLR, despite this trial terminating early (11). The benefits of SRS include that it is an outpatient procedure and may be suitable for those patients unable or unwilling to undergo open surgery. Notable drawbacks are a delay in seizure cessation, post-treatment oedema with subsequent need for steroids, and the unexpected late effects of radiation must be considered.

No papers reported significant declines in verbal or visuospatial memory, intelligence or language. Notably, a published meta-analysis of neuropsychological outcomes criticized the methodological quality of SRS studies, calling for better prospective designed trials (61). A meta-analysis considering SRS published in 2016 reported an average of 14 months to seizure cessation. However, this analysis included studies with small sample sizes (<10 patients) as well as other etiologies, including cavernous malformations (62).

### Radiofrequency

This technique had the lowest seizure-free rate in our mixed effects linear regression model. The quality of evidence for this technique was low, with only one prospective study included in our analysis. Nevertheless, advantages associated with this technique are that it can be performed immediately following SEEG, it has a low risk safety profile and unlike gamma knife there is no latency of effect onset (63). Additionally, according to the same study, there was no need for corticosteroid use following RF-TC compared to SRS. RF-TC is unlikely to be successful as a stand-alone treatment for mesial temporal lobe epilepsy but transient reductions in seizure frequency may predict seizure free outcomes following subsequent open surgery. Such studies were not included in the quantitative analysis within the RF-TC arm as the definitive intervention in these studies was open surgery.

### Laser Interstitial Thermal Therapy

The first reported case of LITT was in 2010. In subsequent years, this technique has shown promising seizure effectiveness. Our mixed-effects model demonstrated overlapping 95% confidence intervals with open surgical models. Visual field defects were the most reported complication in line with previous reports (64). Some of these included visual defects that were asymptomatic to the patient, some of which are not identified until formal visual field testing is done. Such asymptomatic patients might fail UK driving criteria once formal perimetry is conducted. The type of perimetry performed can also skew results, with Esterman perimetry (the current DVLA gold standard) being more lenient. We emphasize that it is crucial to conduct formal visual testing after epilepsy surgery, so that visual complications are not underestimated, and patients can be warned about the potential restrictions on driving. Laser ablation did not show a delayed treatment effect like radiosurgery and its evidence base was more reliable than that of radiofrequency, featuring several more studies. This presents this technique as a strong contender to open surgical methods. Dominant-sided verbal memory decline seemed to be the most common neuropsychological complication but not all studies reported a decline, with an improvement in naming function reported. The much shorter hospital stay associated with LITT compared with ATLR also makes it an attractive first line alternative to open surgery (54). LITT may appear beneficial for post-operative neuropsychological outcome when directly compared with open surgery, perhaps due to relative sparing of the parahippocampal gyrus (50). Our comparative analysis suggested ATLR had the largest effect size estimate of all surgical techniques but overlap of the 95% confidence intervals with SAH and LITT prevents firm conclusions regarding superiority. It is possible, however, that LITT effectiveness is overestimated due to a positive reporting bias associated with novel technologies (halo effect), a reluctance to publish poor outcomes, shorter follow up durations and overcoming of learning curves. A systematic review comparing only the minimally invasive techniques LITT, RF-TC and SRS concluded that LITT shows promising seizure efficacy in the short-term (65). Our linear-regression model results provide a much-needed statistical analysis of the data in comparison to open surgical methods to further support LITT as an emerging technique, but long-term follow ups and direct comparisons are needed to form a firm conclusion. Additionally, we excluded overlapping data from our meta-analysis in order to avoid duplicate cohorts in our statistical analysis.

## Limitations

A notable limitation to the analysis is the varying duration of follow-ups and the significant heterogeneity between studies precluding accurate comparisons. Seizure remissions are known to fall with time and at long term follow-up only around 50% of patients remain free of seizures following open surgery (66). With minimally invasive techniques, comparative follow up rates are shorter, and the durability of such techniques are less well characterized, particularly with SRS where the onset of effect is delayed. Most studies either reported seizure outcome at last follow-up or at pre-determined time intervals. This inconsistency in reporting follow-up times may mask those individuals that are seizure free at their last visit but go on to then subsequently have a seizure.

To mitigate this, we calculated the seizure free rate per-person year. As we do not know the time at which a patient relapses the entire follow up duration is taken as the time at risk. A limitation of this approach is that studies with longer follow up durations have a large number of patient years at risk, which may inflate effect size estimates for novel minimally invasive approaches. Similarly, techniques that result in late seizure recurrences provide the same results as early recurrences, which does not accurately reflect the real-world reduction in seizure burden. Future studies should, therefore, aim to provide more precise data regarding timing of seizure recurrence following surgery.

Another limitation to our analysis is that very few papers were RCTs. Few comparative studies reported whether they were sufficiently powered to detect differences between interventions. Very few studies conducted a prospective calculation of the study size or used blinding to carry out an unbiased assessment of the endpoint. This means that with the small sample sizes present in most publications, studies may have been underpowered to detect statistically significant differences in seizure freedom between interventions. A meta-analysis comparing seizure free outcomes between ATLR, SAH, RF-TC and LITT was recently published which concluded that LITT was significantly less effective than open surgery (67). There are, however, significant methodological differences between our study. The main distinction is that they included studies with 6 months or greater follow up duration. It is widely accepted that open surgical techniques have greatest frequency of seizure recurrence within the first year of surgery (10), incorporating studies with 6 month follow up durations fail to capture this and favor short-term seizure free outcomes. Additionally, the Forest plots and heterogeneity statistics derived from the random effects model between anterior temporal lobectomy and LITT as well as selective amygdalohippocampectomy and LITT were statistically significant (*p* < 0.001). This level of heterogeneity may make such comparisons unreliable.

Only a quarter of the RF-TC papers had neuropsychological outcomes available, limiting our knowledge to one cohort. Additionally, the heterogeneity of neuropsychological tests amongst studies makes it more difficult to draw conclusions. The application of standardized neuropsychological tests administered to those undergoing resective, ablative, and radiosurgical techniques in the future would be of great benefit as it has also been suggested that studies are not using the most appropriate neuropsychological test for the cognitive domain they are testing (68).

Synthesizing complications following open surgery was difficult because of the heterogeneity of complications, no standardized scale for reporting and inconsistency in studies differentiating open surgical techniques (ATLR from SAH). Overall, we found a lower rate of VFDs for open surgery than in the contemporary literature highlighting the lack of formal perimetry testing and a lack of consistency in defining a “clinically significant defect.” Within one review, only one study reported a visual field defect rate of 15%, whilst the rates in the contemporary literature are significantly in excess of this (69).

## Conclusion

Based on effect size estimations of seizure-free rates per person year, there is no evidence to suggest LITT is less effective than open surgical techniques in the short term but long-term outcome data is still lacking. Nevertheless, LITT is becoming a first-line treatment alternative in certain countries as it is more acceptable to patients and if unsuccessful, does not preclude subsequent ablations or open surgery. As with all new technologies, cost and learning curves remain a barrier. Open surgical techniques conveyed similar seizure-free outcomes but may be associated with varying neuropsychological and visual field deficit rates. A randomized control trial is now needed to compare LITT to open surgical methods.

Both SRS and RF-TC were the least effective methods at inducing seizure remission in our synthesis. SRS remains an option for patients that are unfit or do not wish to undergo open surgery. The evidence base for this technique is still limited, the onset of treatment effect is significantly delayed, and adverse effects, notably cerebral oedema, can be associated with severe morbidity.

Secondary outcome measures, such as neuropsychological outcome and intervention-related morbidity, are poorly reported but are important considerations when deciding on first-line intervention. Future studies should also evaluate benefits in secondary outcomes and would undoubtedly benefit from standardization of neuropsychological testing.

## Data Availability Statement

The original contributions presented in the study are included in the article/Supplementary Material, further inquiries can be directed to the corresponding authors.

## Author Contributions

KM, KD, SB, VV, FX, and AA-M contributed to data acquisition and analysis. VV, KM, and JD were involved in the study conception, data interpretation, and drafting the manuscript. AO'K provided statistical expertise. All authors carried out critical review of the manuscript.

## Funding

This research was funded in whole, or in part, by the Wellcome Trust [Grant Number WT - 106882/Z/15/Z]. For the purpose of Open Access, the author has applied a CC BY public copyright license to any author accepted manuscript version arising from this submission.

## Conflict of Interest

The authors declare that the research was conducted in the absence of any commercial or financial relationships that could be construed as a potential conflict of interest. The handling editor declared a past co-authorship with one of the authors JD.

## Publisher's Note

All claims expressed in this article are solely those of the authors and do not necessarily represent those of their affiliated organizations, or those of the publisher, the editors and the reviewers. Any product that may be evaluated in this article, or claim that may be made by its manufacturer, is not guaranteed or endorsed by the publisher.
