# Supplementary material for: Resective, Ablative and Radiosurgical Interventions for Drug Resistant Mesial Temporal Lobe Epilepsy: A Systematic Review and Meta-Analysis of Outcomes
Source: Front Neurol. 2021 Dec 9;12:777845. doi: 10.3389/fneur.2021.777845 (PMC8695716; doi:10.3389/fneur.2021.777845)
Supplement: Supplementary file 1 [file Table_1.DOCX]

Supplementary Information: Search Strategy

Terms used:

(((((selective amygdalohippocampectomy) OR amygdalohippocampectomy) OR SAH)) OR (((anterior temporal lobectomy) OR temporal lobectomy) OR ATLR))) AND ((((epilepsy) AND focal) AND drug resistant) OR refractory);

(focused ultrasound) AND ((TLE) OR temporal lobe epilepsy)

(((TLE) OR temporal lobe epilepsy)) AND ((((((stereotactic radiosurgery) OR gamma knife) OR gamma-knife) OR linear accelerator) OR LINAC) OR cyberknife)

(((TLE) OR temporal lobe epilepsy)) AND ((radiofrequency) OR thermocoagulation)

(((TLE) OR temporal lobe epilepsy)) AND ((((((laser) OR light amplification by stimulated emission of radiation) OR visualase) OR neuroblate) OR LITT) OR laser interstitial thermal therapy
